# Hierarchical cluster analysis of herbicide modes of action reveals distinct classes of multiple resistance in weeds

**DOI:** 10.1002/ps.6744

**Published:** 2021-12-17

**Authors:** Philip E Hulme

**Affiliations:** ^1^ Bio‐Protection Research Centre Lincoln University Christchurch New Zealand

**Keywords:** ACCase inhibitors, cytochrome P450, Jaccard index, photosystem inhibitors, synthetic auxins, target‐site resistance

## Abstract

**BACKGROUND:**

The number of weed species resistant to multiple herbicide modes of action (MoAs) has increased over the last 30 years and may in the future render existing herbicide MoAs obsolete for many cropping systems. Yet few predictive tools exist to manage this risk. Using a worldwide dataset of weed species resistant to multiple herbicide MoAs, hierarchical clustering was used to classify MoAs into similar groups in relation to the suite of resistant weed species they have in common. Network analyses then were used to explore the relative importance of species prevalence and similarity in cluster patterns.

**RESULTS:**

Hierarchical clustering identified three similarly sized clusters of herbicide MoAs that were linked by the co‐occurrence of resistant weeds: Herbicide Resistance Action Committee (HRAC) groups 2, 4, 5 and 9; HRAC groups 12, 14 and 15; and HRAC groups 1, 3 and 22. Cluster membership was consistent with similarities in the physiological or biochemical target of the herbicide MoAs. Network analyses revealed that the number of weed species resistant to two different MoAs was related to the number of weeds known to be resistant to each individual herbicide MoA.

**CONCLUSIONS:**

Hierarchical cluster analysis provided new insights into the risk of weeds becoming resistant to more than one herbicide MoA. By clustering herbicide MoAs into three distinct groups, the potential exists for farmers to manage resistance by rotating herbicides between rather than within clusters, as far as crop, weed and environmental conditions allow.

## Graphical abstract

Schematic representation of hierarchical cluster analyses that are structured by (a) the prevalence of resistant weeds found for different herbicide modes of action (MoAs) and (b) similarity in the biochemical targets of the herbicide MoAs. Each symbol represents a different herbicide MoA, and its size reflects the prevalence of weeds resistant to that MoA.

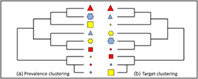

© 2021 The Author. *Pest Management Science* published by John Wiley & Sons Ltd on behalf of Society of Chemical Industry.

## 1 INTRODUCTION

Herbicides are the primary tool for weed management in agricultural settings worldwide yet the repeated use of herbicides with the same mode of action (MoA) has resulted in the widespread evolution of resistance.^1^, ^2^ Accordingly, over the last six decades, the number of herbicide‐resistant weed species has grown dramatically to the point that currently there are >260 weed species known to have evolved resistance to one or more of 167 herbicides belonging to over 20 different MoAs.^3^, ^4^, ^5^, ^6^ This progressive increase in resistant weed species has occurred despite the ongoing implementation of herbicide resistance management strategies since the 1990s that have encouraged an integrated approach, consisting of crop and herbicide rotation with cultural and mechanical weed control.^3^, ^7^, ^8^

The use of herbicide rotations or mixtures within an integrated approach, while potentially resulting in lower levels of target‐site resistance,^3^, ^8^ also runs the risk of promoting nontarget‐site resistance.^9^ Consistent with the increased use of herbicide mixtures and rotations, the frequency with which weed species have been found to be resistant to more than one herbicide MoA also has increased over the last 30 years.^10^ Resistance to several different herbicide MoAs can occur through cross‐resistance or multiple resistance.^11^ Cross‐resistance occurs where plants possess one mechanism, such as a single point mutation in a target‐site gene, which provides the ability to withstand herbicides from different chemical families.^12^ Cross‐resistance also can occur as a result of a single metabolism‐based mechanism that can detoxify herbicides from multiple MoAs. By contrast, multiple‐resistance arises through more than one mechanism, such as combination of both target‐site gene mutation and metabolism‐based resistance conferring resistance to different herbicide MoAs.^13^

The problem of weed species becoming resistant to multiple herbicide MoAs is only likely to get worse because few novel herbicide MoAs have been commercialized in the last two decades^14^, ^15^, ^16^ forcing farmers to rely on an ever more limited range of agrochemical options. It has been argued that increasing rates of resistance to multiple herbicide MoAs over the next 50 years may render existing herbicide MoAs obsolete for many cropping systems.^17^ Yet despite these dire warnings, few predictive tools exist to manage this risk.

To date, current understanding of resistance to multiple herbicide modes of action has stemmed from detailed case studies focused on individual weed species.^18^, ^19^, ^20^, ^21^, ^22^ Resistance to several herbicide MoAs may occur as a result of multiple gene substitution or duplication at the target‐site,^20^, ^23^ but also can occur through nontarget‐site resistance as a result of increased metabolism of herbicides or their reduced translocation and sequestration.^19^, ^20^, ^23^ The nontarget‐site resistance mechanisms are of major concern because they can impart resistance to herbicides from quite different chemical families.^24^ Although case studies are an essential foundation to build a knowledge base for prediction, a quantitative analysis of the patterns in resistance among different herbicide MoAs also could play a key role in identifying more clearly which combinations of herbicide MoAs are least likely to encourage cross‐ or multiple‐resistance. Analyses based on the life‐history traits of weed species can discriminate between resistant and susceptible species, but sample sizes generally are insufficiently large to allow comparisons across herbicide MoAs.^25^ However, the number of records of weeds resistant to more than one different herbicide MoA has increased to the extent thatmachine learning tools can now be used to mine the data effectively and identify emerging properties that may be valuable for more sustainable applications of herbicide rotations and mixtures. Machine learning tools have been used previously to classify active compounds in relation to herbicide MoAs^26^, ^27^ but have not been used to assess the likelihood of resistance to multiple herbicide MoAs.

**Figure 1:**
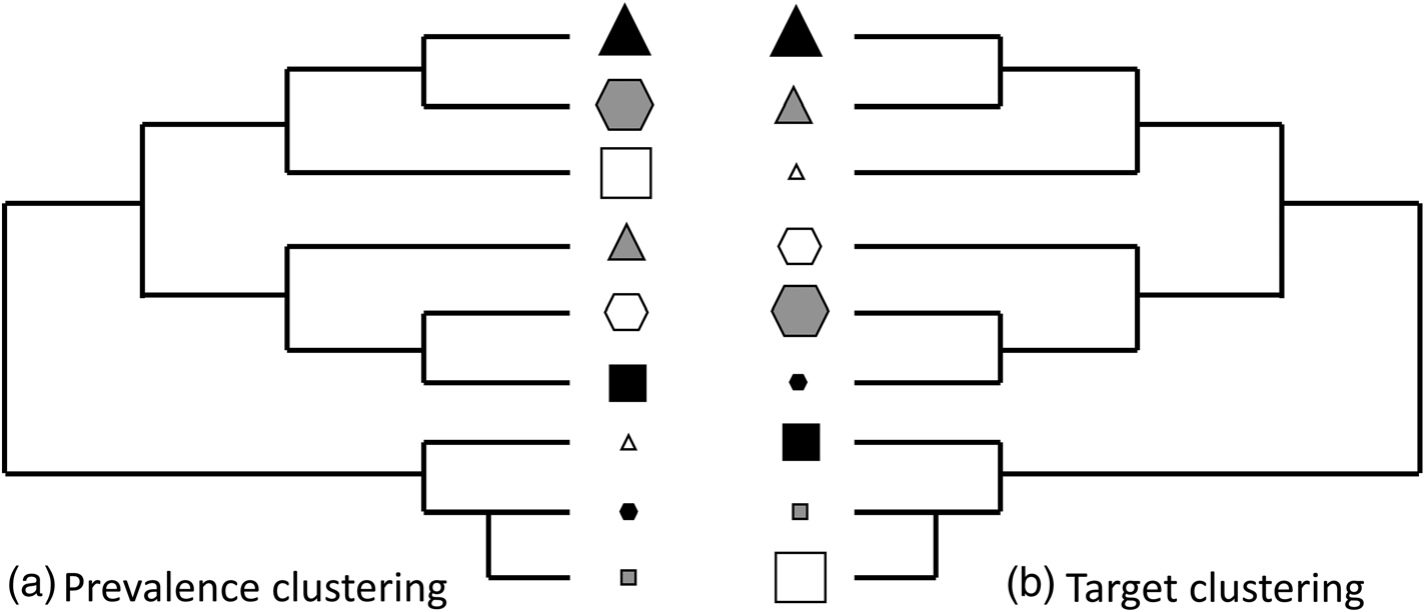
Schematic representation of hierarchical cluster analyses that are structured by (a) the prevalence of resistant weeds found for different herbicide MoAs and (b) similarity in the physiological or biochemical target of the herbicide MoAs. Each symbol represents a different herbicide MoA, and its size reflects the prevalence of weeds resistant to that MoA. The symbol shape describes similar biochemical or physiological targets among different herbicide MoAs while the shading is used to capture the fact that there may be several different herbicide MoAs that share a similar target.

Here, each weed species with known cross‐ or multiple‐resistance is treated as a link between two or more herbicide MoAs. This innovative approach permits machine learning tools to identify hidden structure within the set of weed species found worldwide that are known to be resistant to multiple herbicide MoAs. Hierarchical clustering can be used to classify herbicide MoAs into similar clusters in relation to the suite of resistant weed species they have in common. In hierarchical cluster analyses it is important to consider the role that the prevalence of herbicide‐resistant weeds in each MoA might play in the frequency of resistance to multiple herbicide MoAs. For example, two widely used herbicide MoAs each with many resistant weeds may be more likely to exhibit a greater frequency of cross‐ or multiple‐resistance than two herbicide MoAs that each have few resistant weeds. If the prevalence of herbicide resistance plays a major role in subsequent likelihood of resistance to multiple herbicide MoAs, this suggests the phenomenon is probabilistic. It should therefore be predictable from existing levels of resistance to different herbicide MoAs. Therefore, those herbicide MoAs that have led to many cases of resistant weeds might be grouped together [Fig. 1(a)]. Although a probabilistic explanation might be expected, there also is scope for more subtle associations that do not simply reflect prevalence but instead are shaped by the similarity in the physiological or biochemical target of the herbicide MoA [Fig. 1(b)]. This might reflect commonalities in nontarget‐site resistance mechanisms.

Distinguishing between these two scenarios will help indicate whether the future increase in weeds resistant to multiple herbicide MoAs is inevitable as a result of increasing herbicide use or can be managed by rotating or mixing herbicides with different MoAs. Network analyses can be used to tease apart the role that the number (prevalence) and identify (proximity) of weeds plays in the resulting clusters of MoAs. Combining these analyses should improve the ability to identify herbicide MoAs that are least likely to facilitate the evolution of cross‐ or multiple‐resistance in weeds.

## 2 MATERIALS AND METHODS

### 2.1 Data assembly

Data on the resistance of weed species to one or more herbicide MoAs was extracted from the International Herbicide‐Resistant Weed Database (www.weedscience.org) accessed on 19 June 2021. In an innovative approach to identifying whether resistance to different herbicide MoAs reveals an underlying structure, each weed species exhibiting resistance was assessed as providing a link between two or more herbicide MoAs. The data do not distinguish between cases where a single population of a weed species is resistant to several different MoAs^28^, ^29^, ^30^, ^31^ and where there are multiple populations of a weed species each resistant to only a single, but different, MoA.^32^ Likewise, there is no information as to whether resistance to multiple herbicide MoAs is through cross‐ or multiple‐resistance mechanisms. Thus, the data used here capture the potential for resistance to multiple herbicide MoAs in each weed species and not the mechanism.

Evidence of herbicide resistance was used to generate a binary matrix (resistant or not) of weed species (columns) in relation to different herbicide MoAs (rows). The matrix was consolidated to include only weeds known to have evolved resistance to two or more different herbicide MoAs and only for those MoAs for which ten or more different weed species had evolved resistance. This requirement led to the merging together of the carotenoid biosynthesis inhibitors [Herbicide Resistance Action Committee (HRAC) groups 12, 13 and 27] and resulted in a matrix comprising 101 weed taxa and ten herbicide MoAs. Although these stringent data requirements meant that the analysis focused only on a subset of all currently available herbicide MoAs, the results encompass the herbicides most widely used worldwide.

### 2.2 Hierarchical cluster analysis

Hierarchical cluster analysis is used to identify groups of similar objects, where items in a cluster are more alike than those in different clusters and it is a useful technique for discovering patterns in data that previously may have gone unnoticed.^26^, ^33^, ^34^ The output of a hierarchical clustering algorithm is a dendrogram, which is a 2D tree‐like structure depicting the sequence of nested clusters. Here, this analytical technique is used for the first time to classify herbicide MoAs in relation the frequency with which different weed species have been found to exhibit resistance to two or more different herbicide MoAs. The analysis uses machine‐learning to partition data into a hierarchy of clusters of herbicide MoAs that show high within‐cluster homogeneity and high between‐cluster heterogeneity.^35^ Thus, the resulting clusters should reveal suites of herbicide MoAs that tend to be more strongly linked in terms of resistance in weeds.

An agglomerative algorithm was used to join herbicide MoAs hierarchically where those that were most similar in terms of their associated resistant weed species were joined first. At each iteration, the distance matrix was recalculated using complete linkage clustering. This approach calculates similarity between two clusters by comparing the most dissimilar members (furthest neighbours) and generates compact clusters with a small diameter, even though the clustering can be influenced by outliers.^36^ Cluster dissimilarity was assessed using the Jaccard index because it is appropriate for handling asymmetric binary data and excludes joint absences from consideration while giving equal weight to matches and nonmatches. The optimum number of clusters was determined using the Elbow Method by plotting the agglomerative coefficient against the number of clusters and identifying an elbow in the curve where the rate of change in the agglomeration coefficient declined markedly.^37^ Visual inspection of the cluster dendrogram was used to corroborate the Elbow Method by identifying a set of clusters that were of similar size and avoiding clusters composed of only one item. Hierarchical cluster analysis was undertaken using SPSS v26^38^ on the entire species matrix because attempts to subdivide analyses to contrast patterns between dicotyledonous and monocotyledonous weeds was prevented by there being too many missing distances in the proximity matrix.

### 2.3 Network analysis

Every weed species that is resistant to two or more herbicide MoAs acts as a link between these MoAs and permits the examination of relationships using network tools. The strength of any link between two herbicide MoAs can be estimated by the number of weed species that have evolved resistance to both MoAs. Thus, two MoAs that share many resistant weed species will be found closer together in the network. Network visualization was used to examine whether there was any relationship between the number of weed species resistant to a particular MoA and the linkage‐strength (number of shared herbicide‐resistant weed species) across different MoAs. This network was contrasted with one derived from the proximity matrix generated from the hierarchical cluster analysis in order to assess to what extent network structure was shaped by species prevalence. The ForceAtlas2 layout algorithm as implemented in gephi ^39^ was used for network visualization.

## 3 RESULTS

### 3.1 Broad trends in herbicide cross‐resistance

**Figure 2:**
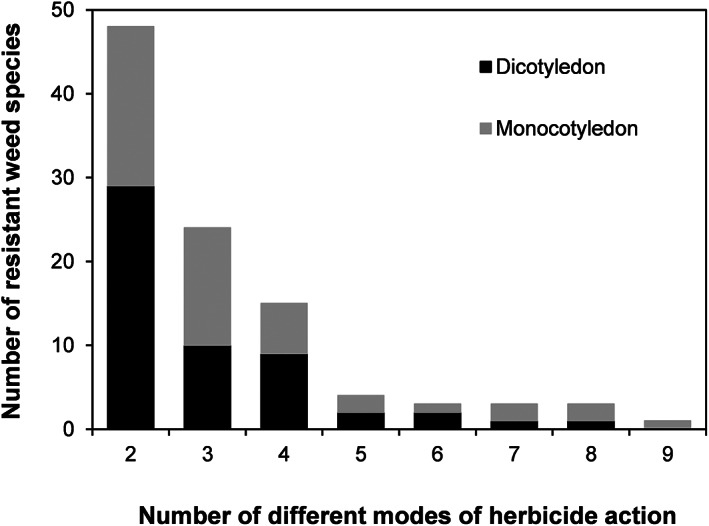
Variation among weed species in the number of herbicide MoAs to which they are resistant. Data are presented for both dicotyledonous and monocotyledonous weeds separately and refer only to the ten different herbicide MoAs examined in the hierarchical cluster analysis.

Of the 263 weed species known to have evolved herbicide resistance, 40% have been recorded as resistant to two or more different MoAs. Although resistance was found to affect all herbicide MoAs, only ten encompassed sufficient weed species for detailed analysis. Considerable variation existed in the number of herbicide MoAs to which different weed species were cross‐ or multiple‐resistant (Fig. 2). Although the median number of herbicide MoAs to which weeds were resistant was three, three grasses (*Lolium rigidum*, *Poa annua* and *Echinochloa crus‐galli*) were resistant to eight or more herbicide MoAs. However, even with these outliers, there was no statistically significant difference between dicotyledonous and monocotyledonous weeds in the median number of herbicide MoAs for which resistance was found (Median test 0.050, *P* = 0.998).

**Figure 3:**
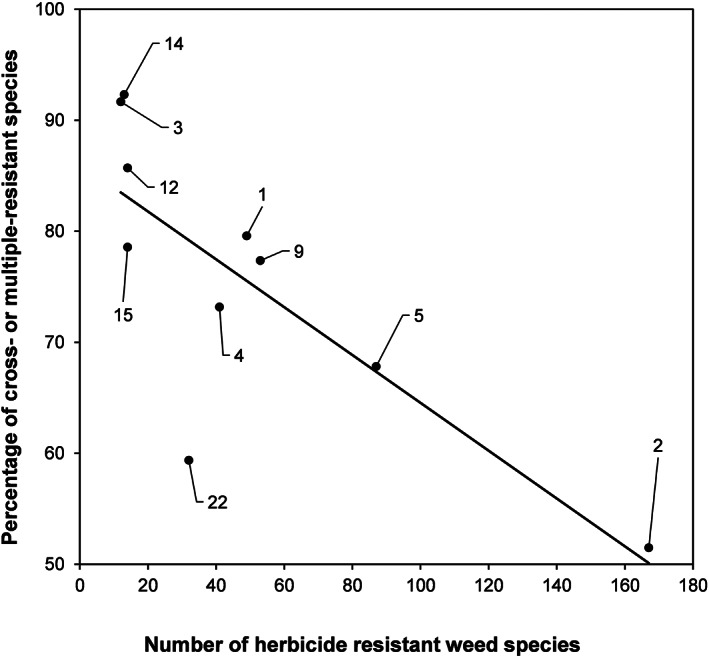
Significant negative relationship (Pearson correlation −0.783, d.f. 8, *P* = 0.007) between the number of herbicide resistant weeds in each of ten herbicide MoA groups and the percentage of those weeds that are resistant to two or more herbicide MoAs. Data points are labelled with the corresponding HRAC MoA classification (see Fig. 5 for the corresponding description of each MoA).

Although herbicide MoAs that had many cases of resistant weed species also had many cases of cross‐ or multiple‐resistance, there was no indication that they were more prone to cross‐ or multiple‐resistance than other MoAs. In fact, there was a significant negative relationship between the number of weed species that had evolved resistance to a particular herbicide MoA and the percentage that subsequently had evolved resistance to one or more additional MoAs (Fig. 3). In some cases, such as for PPO inhibitors (HRAC group 14) and microtubule assembly inhibitors (HRAC group 3), >90% of the weeds that have evolved resistance to these individual herbicide MoAs were resistant to at least one other MoA.

### 3.2 Hierarchical cluster analysis

**Figure 4:**
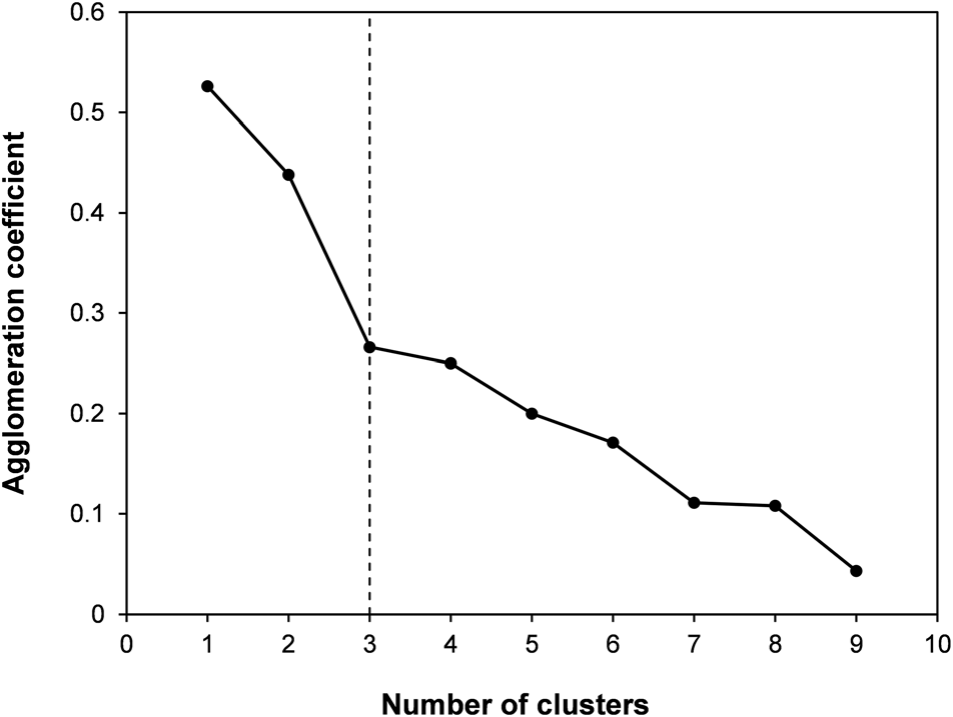
Elbow plot illustrating the rate of change in the agglomeration index as the number of clusters identified increases. The sharpest decline was found between two and three clusters, identifying three clusters (vertical dotted line) as the most parsimonious number to characterize variation in cross‐resistant weeds to different herbicide MoAs.

**Figure 5:**
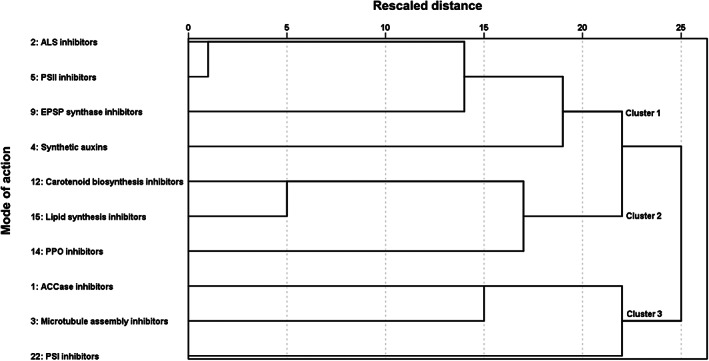
Hierarchical cluster analysis dendrogram identifying three main clusters of resistance to multiple different herbicide MoAs.

Hierarchical clustering identified three similarly sized clusters of herbicide MoAs that were linked by the degree of resistance among them (Fig. 4): Cluster 1 (HRAC groups 2, 4, 5 and 9), Cluster 2 (HRAC groups 12, 14 and 15) and Cluster 3 (HRAC groups 1, 3 and 22). The clusters were of similar size and the height of the dendrogram indicated that Cluster 3 had the largest scaled distance from the other clusters and was the most distinct (Fig. 5). Cluster 1 comprised four of the five herbicide MoAs with the highest number of herbicide‐resistant weed species. The proximity scores highlighted high Jaccard similarity within clusters (Table 1). Although high proximity scores were found between herbicide MoAs with many resistant weeds [e.g. acetolactate synthase (ALS) and photosystem (PS)II inhibitors], similarly high proximities were found for MoAs with few resistant weeds (e.g. lipid and carotenoid synthesis inhibitors).

**Table 1:** Proximity matrix for different herbicide MoAs in relation to their Jaccard similarity in herbicide resistant weed species identities

	Cluster 1			Cluster 2			Cluster 3		
	PSII inhibitors	EPSP synthase inhibitors	Synthetic auxins	Carotenoid biosynthesis inhibitors	Lipid synthesis inhibitors	PPO inhibitors	ACCase inhibitors	Microtubule assembly inhibitors	PSI inhibitors
ALS inhibitors	**0.526**	**0.366**	**0.303**	0.126	0.128	0.140	0.359	0.115	0.154
PSII inhibitors		**0.266**	**0.171**	0.164	0.129	0.164	0.256	0.129	0.238
EPSP synthase inhibitors			**0.246**	0.205	0.182	0.205	0.212	0.156	0.250
Synthetic auxins				0.167	0.108	0.135	0.045	0.051	0.043
Carotenoid biosynthesis inhibitors					**0.438**	**0.200**	0.109	0.211	0.148
Lipid synthesis inhibitors						**0.211**	0.190	0.375	0.111
PPO inhibitors							0.063	0.211	0.107
ACCase inhibitors								**0.250**	**0.160**
Microtubule assembly inhibitors									**0.111**

Bold values represent proximity scores for members within the same cluster.

### 3.3 Network analysis

**Figure 6:**
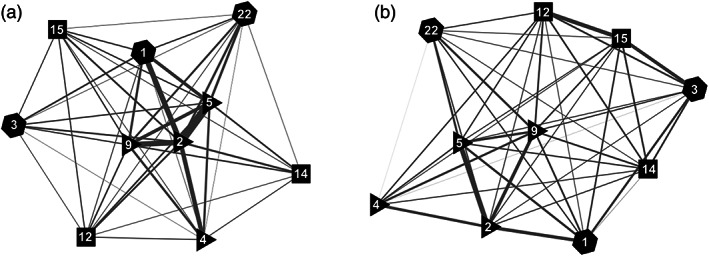
Herbicide MoA networks visualized using the ForceAtlas2 algorithm. (a) The number of species resistant to two linked herbicide MoAs where the width of the links between two MoAs is proportional to the number of species with resistance to both MoAs. (b) Proximity distance between MoAs where the width of the links between two MoAs is proportional to the similarity in weed species composition. The MoAs are described using the HRAC group classification (see Fig. 5 for a description of MoAs) and each cluster is represented by a different symbol: Cluster 1 (triangles), Cluster 2 (squares) and Cluster 3 (hexagons).

Visualization of the herbicide MoA network highlighted the expected relationship that there were stronger resistance links between two herbicide MoAs when these each have many herbicide‐resistant weeds [Fig. 6(a)]. For example, more weeds (167 taxa) have evolved resistance to ALS inhibitors (HRAC group 2) than any other herbicide MoA and many of the strongest resistance links were with other MoAs that also have many herbicide‐resistant weeds (HRAC groups 4, 5 and 9). The proximity network [Fig. 6(b)] presented a more homogenous picture of the associations between different herbicide MoAs but exhibited a different topology to the prevalence network that mapped more closely to the cluster structure. Prevalence was significantly correlated with the magnitude of the proximity score between the different MoAs and explained 54% of the observed variation in proximity scores (*r* = 0.731, d.f. 43, *P* < 0.001).

## 4 DISCUSSION

For the first time, machine learning tools were applied to global data on herbicide‐resistant weeds to extract the previously undescribed structure characterizing weed resistance to multiple herbicide MoAs. This is despite the underlying data describing resistance to different herbicide MoAs being complex and heterogeneous. For example, concerns have been expressed as to the homogeneity, accuracy and independence of classifications of herbicide MoAs as described by the Weed Science Society of America and the HRAC.^40^ Furthermore, a single MoA can encompass multiple different active ingredients that may be applied either pre‐ or post‐emergence and thus differ in the intensity with which they select for resistance.^41^ Finally, the data on herbicide resistance stem from studies across multiple crops, numerous farm management approaches, contrasting climates and many different countries which will all add noise to any underlying signal.^5^ Given this heterogeneity in the underlying data, the emergence of three distinct clusters of herbicide MoAs that are linked by their shared history of resistance indicates fundamental patterns have been captured in the analyses.

Consistent with this view is that the three clusters appear partly to reflect interpretable groupings based on knowledge of herbicide MoAs. For example, herbicides targeting the inhibition of PSI (HRAC group 22) and ACCase (HRAC group 1) are found in one cluster (Cluster 3) and both function through impacts on the lipid components of cell membranes.^42^ Likewise, PPO inhibitors (HRAC group 14) and carotenoid biosynthesis inhibitors (HRAC group 12) found in Cluster 2 act by initiating a self‐sustaining chain reaction of lipid peroxidation which results in leaky cell membranes and a subsequent loss of chlorophyll and carotenoids.^42^ Finally, Cluster 1 includes herbicide MoAs that limit amino acid synthesis by inhibiting EPSP synthase (HRAC group 9) and acetolactate synthase (HRAC group 2) that are important for protein manufacture or for biosynthetic pathways leading to plant growth.^43^ There was no obvious trend that herbicide MoAs targeting monocotyledonous or dicotyledonous weeds were distinctly segregated among the three clusters. For example, dicotyledonous weeds are naturally resistant to ACCase inhibitors^42^ yet while this MoA is associated only with resistant grasses it did not form an isolated cluster.

The tendency of the broad resistance mechanisms of the individual herbicide MoAs to be similar within each cluster is in agreement with the structure of cross‐ or multiple‐resistance being shaped mostly by the similarity in the physiological or biochemical functions of the herbicides. Although cross‐resistance has been shown to occur through multiple amino acid substitutions in the specific target genes,^44^, ^45^, ^46^ this still seems to be a rare occurrence. However, incremental genetic changes at target‐sites will lead to gradually accruing levels of cross‐resistance in weed populations. Several authors have suggested that cross‐ and multiple‐resistance is more likely to occur through nontarget‐site mechanisms.^10^, ^19^, ^47^ Resistance to multiple herbicide MoAs has been attributed to the higher herbicide detoxification activity of cytochrome P450 monooxygenase, glycosyltransferases and glutathione *S‐*transferase in resistant plants.^48^ Likewise, compartmentalization or altered translocation of herbicides away from target areas where they have greatest impact has been shown to confer resistance to different MoAs.^49^ Disentangling the mechanism of cross‐ and multiple‐resistance, whether through target‐ or nontarget‐site mechanisms to determine whether there may be a predictable signal relating to specific herbicide MoAs or to the physiology and biochemistry of weed species remains a challenge. Results of the cluster analysis do indicate the potential for an interpretable structure underpinning resistance to multiple herbicide MoAs. For example, most cases of metabolic detoxification as a mechanism for cross‐ or multiple‐resistance have been found for herbicides that inhibit PSII or ALS,^10^ and these MoAs are found in the same cluster (Cluster 1). However, more detailed analysis is dependent on a greater number of individual case studies and the opportunity to apply broader comparative assessments of underlying mechanisms.^10^, ^48^

Nevertheless, the network analyses also reveal that current patterns in resistance to multiple herbicide MoAs also are likely simply to reflect the prevalence of resistance to individual MoAs. This certainly may be the case for MoAs found in Cluster 1 which brought together four of the five herbicide MoAs with most cases of resistant weeds. Likewise, although the prevalence and proximity networks had different structure, the latter nevertheless still reflected the prevalence of herbicide resistant weeds in the network patterns. This suggests that the progressive evolution of resistance to multiple herbicide MoAs will simply be a matter of time and opportunity, with most cases to date being found among widely used herbicide MoAs that already have accumulated many resistant weeds. In time, however, as particular herbicide MoAs are repeatedly combined either in mixtures or in rotation, the selection for cross‐ or multiple‐resistant biotypes inevitably will increase among a wider range of MoAs. Thus, the use of herbicide rotations or mixtures may be more effective in delaying rather than avoiding resistance.^50^

## 5 CONCLUSIONS

What can be done to mitigate the problem of herbicide cross‐ and multiple‐resistance that has the potential to have major impacts on sustainable agricultural practices and food security? Clearly knowledge of the risk of cross‐ or multiple‐resistance can influence herbicide application strategies. Recommendations to famers regarding less resistant‐prone herbicide options have been made previously using existing data on resistance.^10^ Although this is a valuable first step, the hierarchical cluster analysis points out that simply relying on the frequency with which resistance to two different herbicide MoAs has been observed does not tell the full story. By contrast, cluster analysis possibly provides a broader assessment of resistance risks that pools information across multiple resistant weed species and herbicide MoAs. By clustering herbicide MoAs into three distinct groups, the potential exists for farmers to rotate or mix between clusters and not within clusters, as far as crop, weed and environmental conditions allow. Nevertheless, the cluster analysis included only ten herbicide MoAs, less than half of those currently recognized by HRAC and thus its use for making recommendations to farmers is limited to the most widely used herbicide MoAs for which records of herbicide‐resistant weed species are reasonably common. As more cases of cross‐ or multiple‐resistance emerge, it will be possible to update these analyses to provide increasingly more refined recommendations. A more nuanced set of recommendations can be derived from the proximities between any two different herbicide MoAs. Using herbicide MoAs from different clusters or that have low proximities may even lead to synergies that result in more effective control than either herbicide MoA on its own.^51^ However, communicating such recommendations would require careful thought to ensure the message was clear and did not simply add to the confusion that may exist around herbicide MoA labelling.^52^

## CONFLICT OF INTEREST

The author declares no conflict of interest.

## Data Availability

The data that support the findings of this study are openly available in The International Herbicide‐Resistant Weed Database at http://www.weedscience.org/Home.aspx.
